# Lifting the Sustainability of Modified Pet-Based Multilayer Packaging Material with Enhanced Mechanical Recycling Potential and Processing

**DOI:** 10.3390/polym14010196

**Published:** 2022-01-04

**Authors:** Lynn Trossaert, Matthias De Vel, Ludwig Cardon, Mariya Edeleva

**Affiliations:** 1 Centre for Polymer and Material Technologies (CPMT), Department of Materials, Textiles and Chemical Engineering, Ghent University, Technologiepark, 130, Zwijnaarde 9052, 9000 Ghent, Belgium; Lynn.Trossaert@UGent.be (L.T.); Ludwig.Cardon@UGent.be (L.C.); 2 Laboratory for Chemical Technology (LCT), Department of Materials, Textiles and Chemical Engineering, Ghent University, Technologiepark, 125, Zwijnaarde 9052, 9000 Ghent, Belgium; 3 Eastman Chemical Company, Technologiepark 21-Zone B2, Zwijnaarde 9052, 9000 Ghent, Belgium; Matthias.DeVel@eastman.com

**Keywords:** multilayer packaging material, modified polyethylene terephthalate, co-polyesters, plastic recyclability, hot-fill, transparency, sustainability

## Abstract

Sustainability and recyclability are among the main driving forces in the plastics industry, since the pressure on crude oil resources and the environment is increasing. The aim of this research is to develop a sustainable thermoformable multilayer food packaging, based on co-polyesters, which is suitable for hot-fill applications and allows for recycling in a conventional waste stream. As a polymer material for the outer layer, we selected a modified polyethylene terephthalate (PETM), which is an amorphous co-polyester with a high glass transition temperature (±105 °C) and thus high thermal stability and transparency. The inner layer consists of 1,4-cyclohexylene dimethanol-modified polyethylene terephthalate (PETg), which is allowed to be recycled in a PET stream. Multilayers with a total thickness of 1 mm and a layer thickness distribution of 10/80/10 have been produced. To test the recyclability, sheets which contained 20% and 50% regrind of the initial multilayer in their middle PETg layer have been produced as well. The sheet produced from virgin pellets and the one containing 20% regrind in the middle layer showed no visible haze. This was not the case for the one containing 50% regrind in the middle layer, which was confirmed by haze measurements. The hot-fill test results showed no shrinkage or warpage for the multilayer trays for all temperatures applied, namely 95, 85, 75 and 65 °C. This is a remarkable improvement compared to pure PETg trays, which show a visible deformation after exposure to hot-fill conditions of 95 °C and 85 °C.

## 1. Introduction

Plastics have become the number one material for many applications since World War II, starting with rather cheap bulk materials, and delivering more and more high-quality niche products during recent decades [1,2,3,4]. This has led to a total annual production of plastics that reaches over an astonishing 330 million tons [5]. One of the main plastics applications is food packaging. This is due to the good barrier properties, low cost, good processability and versatility of plastic packaging, as well as their lightweight nature, which makes them easy to handle [6]. Bulk polymers offer the possibility to pack foods in a controlled environment for a certain amount of time. However, for some products, e.g., fruit juices, dairy products and ready-meals, a sterile environment is required which is created by heating up these products and the polymeric package to temperatures near 100 °C and filling in the package at these elevated temperatures. This process is industrially known as ‘hot fill’ [7,8].

To provide sufficiently hermetic environments, some food products are stored in a multilayer package composed of a wide range of polymers [9,10]. This multilayer structure results in recycling difficulties and might lead to more waste that is incinerated or landfilled. Lack of recyclability also implies a higher need of ‘virgin’ material, either crude-oil-based or biobased, which results in an even larger pressure on the environment [5,11]. It is obvious that there is a need for thermally stable materials that are, in addition, easy to be recycled.

One of the materials that is used for hot-fill purposes nowadays is crystalline polyethylene terephthalate (CPET). The crystallinity of the material creates good thermal stability [12]. However, this comes with the drawback of a lack of transparency, which is often required in food applications. Another frequently used thermally stable material is polypropylene (PP), due to its strength and high chemical resistance. As is the case for PET, PP has a high melting temperature, making it suitable for hot fill and use in a microwave oven [13]. Aside from the lack of transparency of the crystalline polypropylene, the material suffers from poor O_2_ and CO_2_ barrier properties. To counter the oxidation of packaged food, multilayers with ethylene vinyl alcohol (EVOH) are often applied, making the product very difficult to be recycled [14]. Furthermore, polystyrene has been applied for hot fill due to its high glass transition temperature of about 95 °C, and this is extensively used for packaging dairy products, salad bowls, fresh meat and fast foods [15]. The material has the additional advantage that it can be made highly transparent [6]. Despite these good properties, awareness is growing as to the possible health issues that are related to residual styrene, which is a mutagenic component, migrating from the package into the food [6,16]. One of the strategies to enhance thermal stability is the application of multilayer materials with outer layers produced from polymers with a high glass transition temperature [17] or layers with inorganic fillers [18]. Evidently, such multilayer materials lack recyclability as the separation of different layers requires sophisticated experimental techniques [19,20] that are currently unavailable in industry.

PET-based multilayer materials have been attracting growing interest during recent years [9,21,22,23,24,25,26]. Notably, recently developed co-polyesters might lead to solutions for the problems related to the previously mentioned polymeric materials and provide good thermal properties for final products [27,28] as they are capable not only of thermal but also of microwave-assistant sterilization [21,29]. Modified polyethylene terephthalate (PETM) from Eastman Chemical Company is particularly interesting, because of its high thermal stability, allowing for the hot-fill applications, as well as the possibility to combine it with PET in recycling streams. Moreover, co-polyesters typically feature high transparency, which also might be a benefit for food packaging purposes. It should be noted that PET-based multilayer materials have high recycling potential, as suggested by a number of recent studies on the recyclability and development of the sustainable packaging [30,31,32,33]. Despite the abovementioned extensive studies, a demand exists for (i) thermally stable (capable of hot fill applications), (ii) recyclable and (iii) transparent materials.

**Figure 1:**
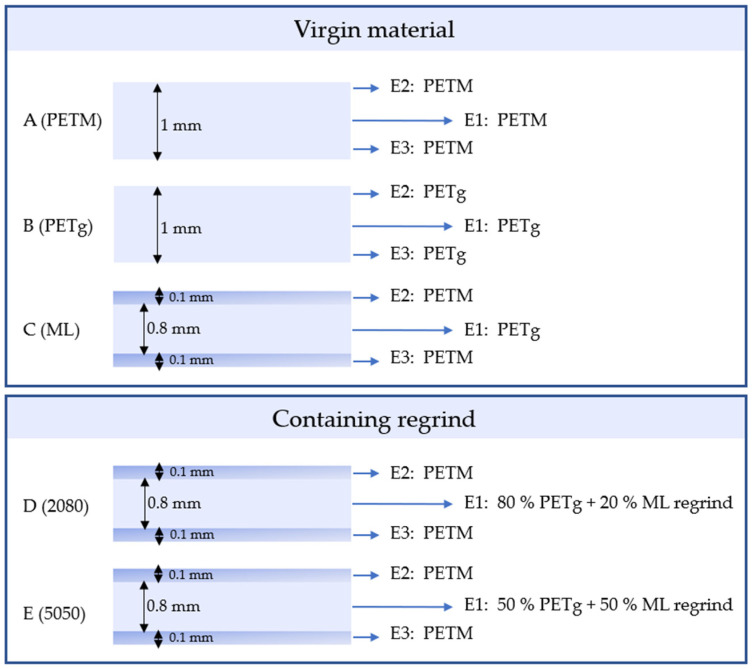
Composition of the produced multilayer sheets in this work, with a relative layer thickness distribution of 10/80/10. (**A**,**B**): pure sheets (PETM, PETg), (**C**): multilayer (ML), (**D**,**E**): the multilayer containing 20% regrind (2080) and 50% (5050) regrind in the middle layer.

In this work, we produced qualitative thermoformable and transparent multilayers which contain polyethylene terephthalate glycol (PETg) as the inner layer and PETM as the outer layers, ensuring the dimensional stability at high temperatures during hot fill. As we aimed at high recyclability, we also prepared multilayers which contained regrind material from the previously produced multilayers inside their middle layer. The variety of these manufacturing approaches is illustrated in Figure 1. The influence of the extrusion residence time during the preparation of the multilayer material on the degradation of the extrudate was investigated to obtain the optimal processing conditions for this type of polyesters. For the final multilayer material, thermoforming and hot-fill experiments were performed, showing the remarkable thermal stability of the produced packaging materials. To test the applicability of the recycled material, we performed haze measurements as well. It is shown that multilayers with up to 20% recycled polyester in the inner layer show sufficiently good transparency.

## 2. Materials

For the production of the multilayer packaging material, 1,4-cyclohexylene dimethanol (CHDM)-modified polyethylene terephthalate (PETg, Eastar EN076, of which up to 10% of the ethylene glycol is replaced by CHDM) and the thermally stable co-polyester PETM provided by Eastman Chemical Company have been considered.

PETg has a processing melt temperature between 277 °C and 293 °C. PETM has a processing melt temperature between 250 °C and 298 °C.

## 3. Methods

### 3.1. Extrusion-Based Sample Preparation with Variable Residence Time

The influence of the processing conditions was tested via the preparation of “residence samples” with different residence times in a Thermo Scientific HAAKE Minilab twin-screw extruder. This equipment enables the testing of different residence times by keeping the material inside the machine for an additional period. The residence time without the added time is 4 min. For optimal operation of the equipment, the loaded volume of the polymer had to be between 7 and 7.5 mL. The exact masses that were used were 9.6 g for PETg and 9 g of PETM. Before extrusion, the pellets were dried in a FarragTech CARD 40E. PETg and PETM were, respectively, dried at 120 °C and 60 °C overnight, and afterwards put in a lab oven at 80 °C.

The temperature of the instrument was set at 270 °C and 290 °C for PETM and PETg, respectively. The added residence time was varied between 2 and 11 min in steps of 3 min for both pure PETg and PETM, as shown in Table 1. After the preset residence time was reached, the polymer was let out of the extruder for a total 1 min of extrusion (cooling down to room temperature by ambient air of 20 °C), then the extrudate was cut off to obtain a representative sample for executing the measurements, as described below. The samples are referred to as described in Table 1.

### 3.2. Extrusion-Based Production of Sheets

A pure PETM sheet; a pure PETg sheet; a multilayer produced from virgin pellets (PETM/PETg/PETM, Figure 1, sample C) and multilayers containing, respectively, 20% (Figure 1, sample D) and 50% (Figure 1, sample E) regrind of the first multilayer in the middle layer, were produced with a layer thickness distribution of 10/80/10 and a total thickness of 1 mm. Before processing, the polyester pellets were placed in a Drylogic II D100H or in a FarragTech CARD 40E dryer. The suggested drying time for both Eastar EN076 and PETM was 4 to 6 h. The suggested temperature was 150 °C and 85 °C, respectively.

The polymer sheets and multilayers were extruded with a LabTech co-extrusion unit. Three single-screw extruders were used, all with a length-over-diameter ratio (L/D) of 30. The inner layer and one outer layer were extruded using a single crew extruder with a 30 mm diameter. The other outer layer was extruded using a single-screw extruder with a diameter of 20 mm. To obtain outer layers with a thickness of 1/10 of the total thickness and an inner layer of 8/10 of the total thickness, the rotational speed of the screws were set at 80 rpm for the middle layer (E1), and 10 and 40 for the larger (E2) and smaller extruders (E3) used for the outer layers, respectively.

The layer thickness distribution was not of importance for the sheets made from layers from the same material. For the PETg sheet production, a more evenly distributed rotational speed was used for optimal material extrusion, namely, 48 rpm for the largest extruders and 67 rpm for the smaller one. The temperatures in the barrel and the screw speed can be found in Table 2 for each sample. The table also shows the abbreviations which will be used for the pure sheets (PETM, PETg), the multilayer (ML) and the multilayers containing 20% (2080) and 50% (5050) regrind in the middle layer.

### 3.3. Thermoforming of Sheets

For thermoforming, Formech 508FS equipment was used. The sheet was heated above its glass transition temperature (*T*_g_) by heated ceramic blocks, which can be drawn over the sheet. The intensity of the heating of the ceramic blocks, both in operation and in stand-by mode, can be chosen, as well as the heating time. The temperature was measured with a portable infrared temperature measurer. In Table 3, the temperature ranges which were used for each type of sheet are given.

**Figure 2:**
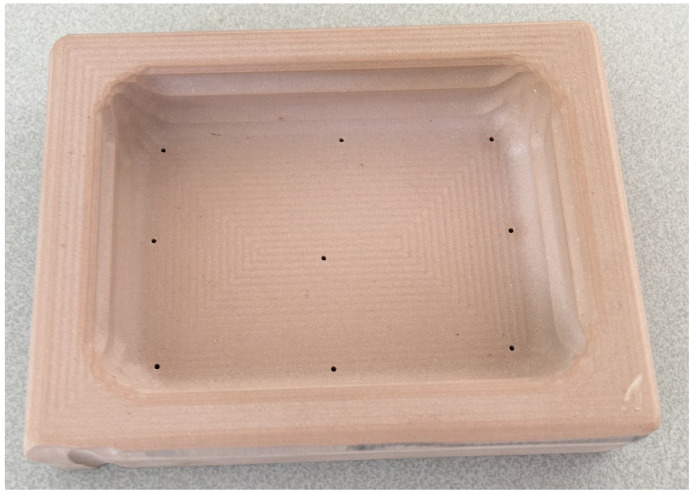
Ceramic tray mold used for thermoforming the PETM, PETg and multilayer sheets.

The mold for thermoforming was an additive manufactured ceramic tray mold (Figure 2). It produces trays with a depth of 19 mm, a top surface of 58 by 82 mm and a bottom surface of 48 by 72 mm.

### 3.4. Hot-Fill Test

The hot-fill test is carried out by filling the thermoformed trays produced from the abovementioned materials (Table 1) with water at specific temperatures. The test is carried out at temperatures of 65, 75, 85 and 95 °C. The hot water is kept inside the tray for 2.5 min. Afterwards, the tray is quenched in a water bath at 20 °C for 1 min. This method is based on the test method from the International Society of Beverage Technologists [34] but deviates from it since the trays are not sealed after hot fill, which means that the content of the tray is not exposed to the hot-fill vacuum [35,36]. However, this test already gives a valid indication of the resistance of the material against filling temperatures used in industrial applications for sterilization.

The heat resistance is determined by controlling the dimensional stability of the tray. The change in volume of the tray is determined by filling the cup with water and weighing the amount of water which can be filled in the tray. Dividing the mass of the water by its density gives the volume of the tray. This is done both before and after hot filling. This way, a percentual difference in volume can be calculated using the following equation:

(1) $$change\;in\;volume=\frac{V_{0}-V_{hot\;filled}}{V_{0}}\times 100\%$$

### 3.5. Analytical Techniques

#### 3.5.1. Differential Scanning Calorimetry (DSC)

DSC was performed with the NETSCH DSC 214 Polyma under an inert N_2_ atmosphere, with a gas flow of 40 mL min^−1^. Aluminum sample pans were used, in which 10 mg sample was placed. Two heating runs and one cooling run were carried out, with a heating rate of 10 °C min^−1^ between 20 °C and 300 °C. The degree of crystallinity can be estimated as follows:

(2) $$\chi_{c}=(\Delta H_{m}-\Delta H_{cc})/\Delta H_{m}^{*}\times 100\%$$

in which $\Delta H_{m}$ is the melt enthalpy, $\Delta H_{cc}$ is the cold crystallization enthalpy and $\Delta H_{m}^{*}$ is the melt enthalpy for a 100% crystalline material. Since the crystalline fraction in PETg and PETM consists of PET segments, the melt enthalpy for a 100% crystalline PET material [4,37], which is 140 J g^−1^, is used in the calculations.

#### 3.5.2. Simultaneous Thermal Analysis

Thermogravimetric analysis (TGA) and oxidative initiation temperature (OIT) analysis were performed by means of a simultaneous thermal analyzing instrument (STA) on the NETZSCH STA 449 F3 Jupiter. The analysis was performed under an O_2_ atmosphere with a temperature gradient of 10 °C min^−1^ ranging from 40 °C to 500 °C. The gas pressure of O_2_ was set at 50 mL min^−1^ and the pressure of the protective gas (N_2_) was set at 20 mL min^−1^.

#### 3.5.3. Fourier Transform Infrared Spectroscopy

Attenuated total reflection Fourier transform infrared spectroscopy (ATR FTIR) was carried out with the Brucker Tensor 27. The spectral range was from 4000 to 600 cm^−1^, averaging 32 scans per measurement. Before the analysis, the samples were dried for 4 h in a lab oven at 80 °C.

#### 3.5.4. Color and Haze Measurement

Color and haze measurements were performed with the Hunterlab UltraScan VIS equipment. The haze measurement was based on the ASTM D1003 standard; however, it deviated in terms of the instrument’s geometry [38]. The sensor was set to ‘*total transmission*’ mode and the ‘*haze*’ method was chosen. Haze was calculated with the following equation:

(3) $$Haze=\frac{Y_{Diffuse\;transmission}}{Y_{Total\;transmission}}\times 100\%$$

For the illuminant/observer combination, the ‘C/2°’ option was chosen. The *X*, *Y* and *Z* tristimulus values, related to the CIE color space, enabled the determination of the yellowness index (*YI*) according to the ASTM E313 standard, using following equation [39]:

(4) $$YI=100\times \frac{C_{x}\times X-C_{z}\times Z}{Y}$$

in which *C_x_* and *C_z_* are constants which equal 1.2769 and 1.0592, respectively, when the C/2° measuring mode is used.

#### 3.5.5. Inherent Viscosity Measurement

Inherent viscosity *η*_inh_ was measured with a Viscotek Y501C Differential Viscometer, a two-capillary relative viscometer and a DSV Autosampler. A sample with a mass between 0.118 and 0.133 g was dissolved in propylene glycol monomethyl ether (PM95) at 115 °C and cooled down. The inherent viscosity was determined through a comparison with the pure solvent and is defined as the ratio of the natural logarithm of the relative viscosity ($\eta_{r}$) to the mass concentration (c) of the polymer in the solvent:

(5) $$\eta_{inh}=\frac{\ln\left(\eta_{r}\right)}{c}$$

## 4. Results and Discussion

In this section, an interconnected discussion is provided, starting with the results for the samples with varying residence times inside the extruder to define the influence of degradation at specifically longer residence times. This includes the interpretation of the results of FTIR, viscosity, DSC and TGA measurements. This is followed by a discussion of the influence of thermoforming on the degradation of the material by an analysis of FTIR-, viscosity-, DSC- and haze-measurement results. Lastly, the results of the hot-fill tests on the different trays are presented.

### 4.1. Degradation during Extrusion

**Figure 3:**
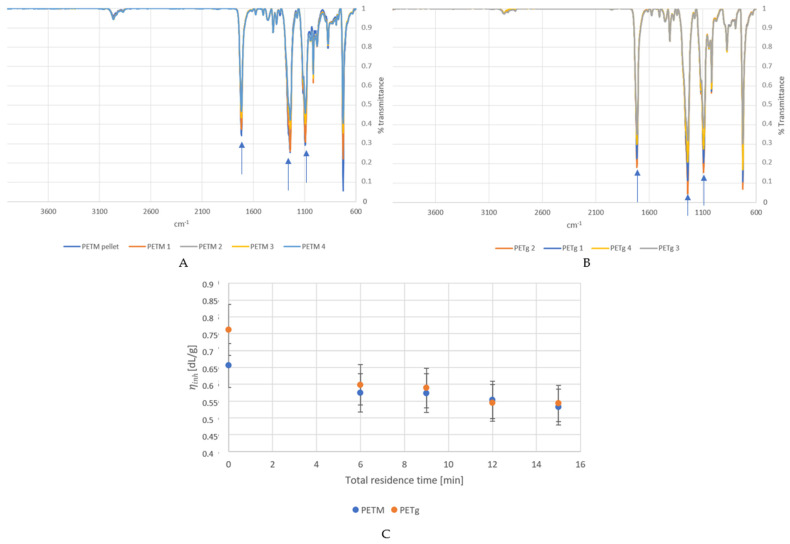
FTIR spectra for the virgin PETM (**A**) and PETg (**B**) (top curves) samples and residence time samples with various total residence times (see Table 1). Arrows are pointing at the increasing intensity of the C=O stretching vibration band ν_C=O,st_ = 1716 cm^−1^ relative to the C-O-C stretching band ν_C-O-C,st_ = 1240 and 1093 cm^−1^. (**C**) Inherent viscosity $\eta_{inh}$ of the PETM and PETg residence time samples (see Table 1) vs. total residence time.

To study the influence of the processing conditions we prepared PETg and PETM samples with various residence times in the extruder (Table 1). We aimed to check for extrusion-induced degradation of the polymer material. For the samples with various residence times, we checked for the formation of new reaction products by FTIR and chain length changes through inherent viscosity measurements. The results are shown in Figure 3. The FTIR spectrum of the PETg pellet is not included in the graph, because of its high crystallinity, leading to a spectrum which was substantially different from the amorphous extruded samples.

FTIR analysis (Figure 3A,B) showed no new peaks for samples with higher residence times (up to 15 min) for both PETM and PETg, which means that no new functional groups were formed. However, the intensity of the peak at 1716 cm^−1^ (related to the C=O stretching) relatively increased compared to those at 1240 and 1093 cm^−1^ (related to C–O–C stretching) at longer residence times. This could be related to chain scission at the ester bonds, which reduces the 1240 and 1093 cm^−1^ signals, and an additional oxidation, leading to more C=O bonds. Chain scission is promoted in the extruder, due to the high shear and temperatures which are present [40]. Moreover, the presence of residual moist in the pellets can lead to hydrolysis of the ester bonds. However, the effect seemed to stabilize starting from residence times of 9 min.

Figure 3C shows the inherent viscosity vs. residence time in the extruder, with a similar trend. For both PETM and PETg, $\eta_{inh}$ decreases as after processing (decrease of 12.4% for PETM and 21.4% for PETg when the virgin pellet is compared to the first residence time sample), which means that the residence inside the extruder leads to chain scission and a decrease in the polymer’s average chain length. The change in $\eta_{inh}$ between the different residence times was rather insignificant, meaning that a longer residence time does not significantly affect the degradation through chain scission. The higher degradation of the PETg pellet compared to the PETM pellet could be due to the higher temperatures which were used during extrusion (270 °C for PETM compared to 290 °C for PETg).

### 4.2. Crystallinity during Extrusion

As crystallinity determines the transparency and mechanical properties of the polymer sheets, we studied the influence of the processing conditions (residence time) on the crystallinity of the pure PETg and PETM samples reported in Table 1 via DSC.

**Figure 4:**
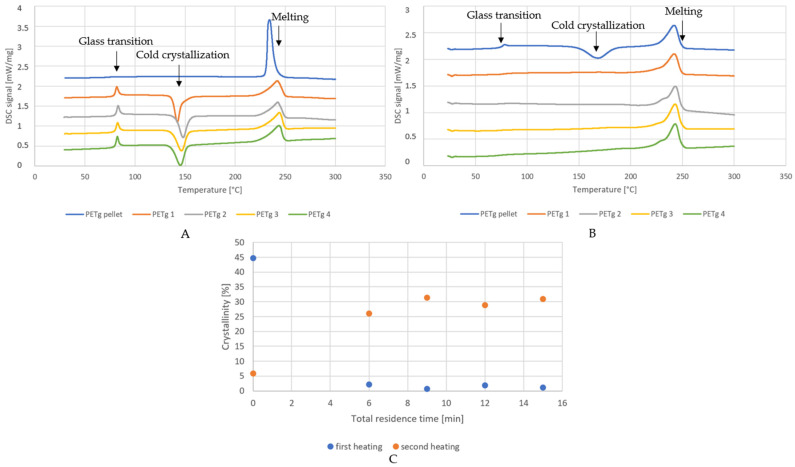
DSC curves of the first (**A**) and second (**B**) heating run of the PETg samples. Crystallinity of the PETg residence time samples vs. total residence time (**C**) (Table 1).

As expected, no crystallinity was detected for the PETM samples, since this is a fully amorphous polymer. The crystallinity results of the PETg samples is shown in Figure 4. The crystallinity of the PETg extrudates was low (less than 2.5%), according to the results of the first heating run. This is due to the fast cooling of the thin extrudate by air at room temperature after extrusion. PETg can thus be extruded with very low crystallinity.

The second heating run in DSC, which is related to the material itself (and is not influenced by the thermomechanical history), showed that the crystallinity increases if the residence time is longer. The difference was especially significant between the virgin pellet and the first residence time sample. This was probably caused by a much slower crystallization of the PETg pellet with longer chains and thus higher average chain lengths. For the extrudates, the PETg had degraded to a certain degree (thermally and through hydrolysis), as also observed in the inherent viscosity measurements, which leads to shorter chains and smaller average chain lengths. This effect was investigated by Pirzadeh et al. [41] and Mancini et al. [42] as well, and was found to lead to higher crystallinity of the material. This is caused by the fact that shorter chains can move more easily, which makes it easier for them to organize themselves in crystalline regions. Moreover, chain scission results in a decrease in the number of entanglements, which stimulates crystallization as well (Oromiehie et al.) [43]. The experimental data for our samples are consistent with those observations. Again, the effect seems to stabilize at residence times of 9 min.

### 4.3. STA Analysis for Extrusion

**Figure 5:**
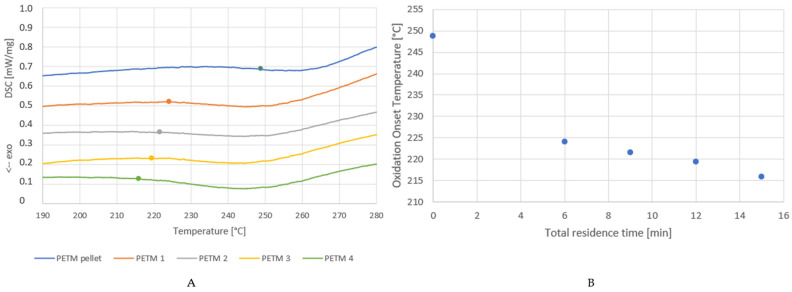
(**A**) DSC signal of the STA analysis of the PETM residence time samples (Table 1) for the determination of the oxidation onset temperature (dots). (**B**) Oxidation onset temperature vs. residence time for PETM samples.

The oxidation initiation temperature was measured by analyzing the DSC signal of the TGA instrument. Since oxidation is an exothermal process, the OIT could be defined as the first drop in the signal. In Figure 5, the relevant part of the DSC curves for the PETM residence time samples is shown. The dots represent the respective OITs. It is clear that the OIT is lower if the sample has been exposed to a longer residence time in the extruder. The difference between the virgin pellets and the processed samples is the most striking. Das et al. [44] found that the OIT of unprocessed PET in an air atmosphere (21% oxygen) with a heating rate of 10 °C min^−1^ was 374 °C. For the PETM pellet, the OIT is 244 °C under a 100% oxygen atmosphere.

As shown in Figure 5B, the OIT decreases as the total residence time increases for the PETM samples. The shape of the OIT vs. total residence time curve is similar to that of the inherent viscosity vs. residence time curve, meaning that for the samples with shorter polymer chains the OIT is lower. This could be due to the presence of a greater number of chain-end groups, which are prone to oxidation as polymer chains become shorter after degradation at longer residence times. As more chain ends are present, and oxygen has more easily accessible sites to attack functional groups prone to oxidation (alcohol groups, for instance). Shorter chains also lead to a higher chain mobility, which results in a higher reactivity.

**Figure 6:**
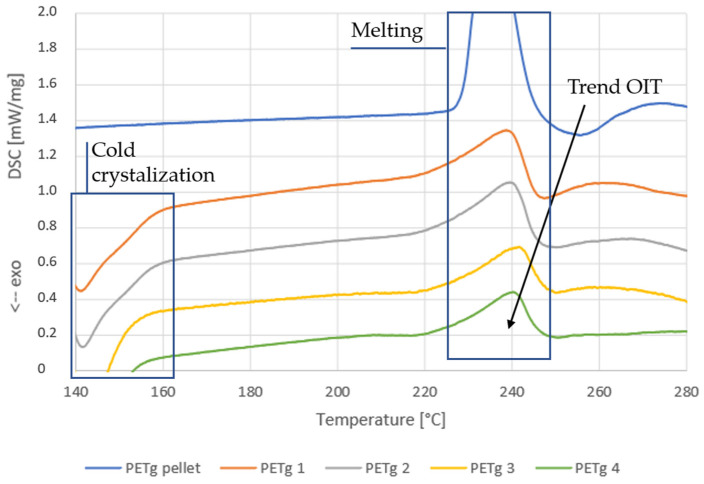
DSC curves of the STA analyses of the PETg residence time samples, extruded with the HAAKE Minilab twin-screw extruder, for the determination of the oxidation initiation temperature.

For PETg (Figure 6), the DSC signal was more complex. This is because PETg is semi-crystalline, which results in an exothermal cold crystallization peak (left side of the graph) and a melt peak. It is visible that there was a drop in the signal just after melting. This means that the OIT occurred at approximately the same time or just after melting occurs, which makes it impossible to define the OIT properly. Although no OIT could be determined, a slight trend is visible. For the pellet, a large drop is situated after the melt peak. However, this drop becomes smaller when the residence time increases, and the melt peak seems to be smaller when the residence time increases. This could mean that the OIT moves to lower temperatures and has a larger overlap with the melt peak at longer residence times. This exothermal process partially compensates for the melt peak, resulting in a smaller endothermal peak. The decreasing trend of OIT for longer residence time samples of PETg is similar to that of PETM samples.

**Figure 7:**
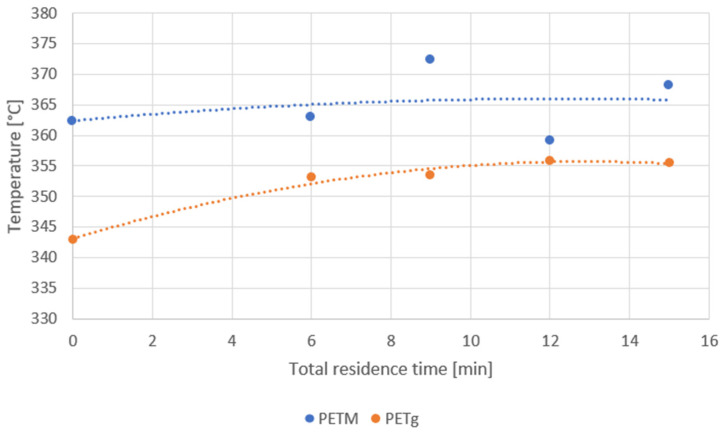
Temperature at which the residence time sample, extruded with the HAAKE Minilab twin-screw extruder, lost 5% of its mass, with the dashed lines as a guide for the eye.

To obtain an idea of the thermal stability of the polymer samples, the temperatures at which the residual mass of the sample is 95% of the initial mass are depicted in Figure 7. For PETM, the sample lost 5% of its mass between 359 °C and 372 °C, and PETg lost 5% between 343 °C and 356 °C.

For PETg, a clear trend is visible (orange symbols, Figure 7). The longer the residence time, the higher the temperature at which the sample lost 95% of its mass. A possible explanation is that, due to oxidation during processing, structures were formed which were more stable than the original polyester chains, leading to slower thermal degradation of the sample. Notably, the thermal stability of the virgin pellets was significantly smaller than for the residence time samples, as the 5% mass loss temperature differed by 3%.

For PETM, no clear trend can be seen (blue symbols, Figure 7). It is probable that the deviation in the measurements of the PETM samples was too large and these results cannot be used to draw conclusions on the change in behavior depending on the residence time. The difference between residence time samples and virgin pellets is insignificant as well.

Figure 7 shows that the thermal stability of PETM is larger than is the case for PETg. Thompson et al. [45] attributed the higher thermal stability of modified PET samples compared to regular PET to the larger number of alicyclic units in the chain. This reasoning also applies to our case, since PETM is modified to a larger extent with cyclic units than is the case for PETg (modified with CHDM). They carried out TGA on PET, which was modified with bicyclohexyldimethanol under a nitrogen atmosphere, giving a 5% mass loss temperature at 390 °C. This is somewhat higher than was the case for the PETg and PETM tested in this work. This is probably due to the 100% oxygen environment which was used in our case, which enabled not only thermal degradation, but also thermo-oxidative degradation reactions.

### 4.4. Degradation Induced during Thermoforming

The thermoforming process can induce polymer degradation as well. To obtain an insight in that process, we performed FTIR and inherent viscosity measurements.

Since only the outer layer is detected by the FTIR analysis in its reflectance mode, the multilayers have a spectrum which is similar to the spectrum of the PETM sheet and no conclusions can be drawn for the inner PETg or regrind layer. For all the sheets, it was visible that no new peaks were formed in the spectra of the thermoformed samples. Thus, no new functional groups were formed, and no severe degradation had occurred. The spectrum did not change notably after thermoforming, which means that the degradation was negligible. However, for the PETg sheet, the peaks related to C-O-C stretching went down relatively to the peak of C=O. This can be explained in the same manner as was done for the residence time samples and means that PETg seems to be more prone to degradation.

**Figure 8:**
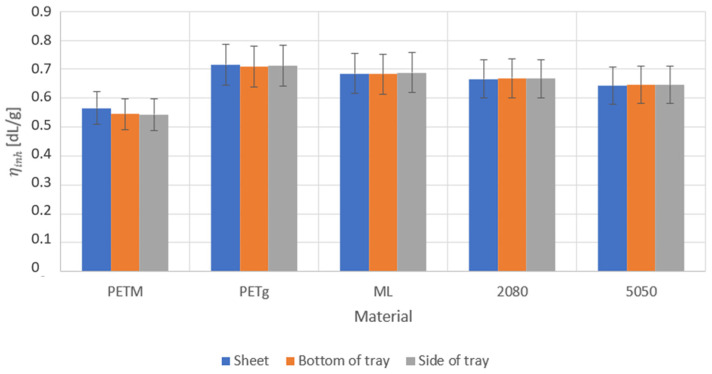
Inherent viscosity of the sheets and multilayer sheets before (blue) and after thermoforming for the samples taken in the bottom (orange) and at the wall (gray) of the thermoformed tray. All the samples are listed in Table 2.

$\eta_{inh}$ measurements, as shown in Figure 8, allow us to conclude that the thermoforming process induced little to no thermal degradation as the inherent viscosity stayed the same before and after thermoforming for all the materials. The PETM sheet had a lower viscosity than the PETg sheet. This was not expected from the results from the processed residence time samples, which show that the viscosity of PETM and PETg did not significantly differ after processing (Figure 3B). The viscosity of the PETM sheet was possibly lower because the PETM pellets were not fully dry during processing, which led to hydrolysis of the ester bond and thus led to shorter chains. The $\eta_{inh}$ of the virgin multilayer was comparable with the $\eta_{inh}$ of the PETg sheet. If the multilayers are compared, there was no significant decrease in the $\eta_{inh}$, and thus the (average) chain length, meaning that the additional processing step of the regrind did not cause additional degradation through chain scission.

### 4.5. Crystallinity of the Sheets and Trays

**Figure 9:**
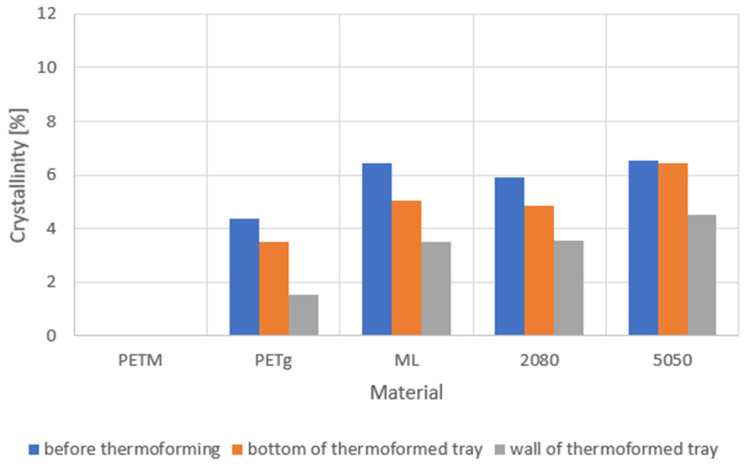
Crystallinity of the sheets and multilayer sheets (Table 2) before (blue) and after the thermoforming process for the samples taken in the bottom (orange) and in the wall (gray) of the tray.

As polymer sheets were heated up to between 135 °C and 165 °C in the thermoforming process, the crystallinity of the polymers in the final package mold can be different from that of the unmolded sheets. Furthermore, the bottom part and the walls of the thermoformed tray were subjected to different stresses, meaning that the crystallinity and thus transparency and mechanical properties can be different. To obtain an insight into this, we measured the effect of thermoforming on the crystallinity of the samples from the walls and bottom of the thermoformed trays and compared this to the crystallinity of the non-thermoformed samples (Figure 9). It is important to note that the crystallinity in the multilayers is totally caused by the PETg fraction, since PETM is a 100% amorphous material, and that the percentages of crystallinity shown in Figure 9 are related to the total mass of the samples and not only on the PETg fraction.

From Figure 9, it can be concluded that the largest crystallization occurred in the multilayers. The amounts of crystallinity for all multilayer types (with and without regrind) are comparable. However, the ‘5050’ sheet appeared more hazy, which means that the additional haziness was not caused by crystallinity, but might be caused by the presence of water during extrusion, which formed voids. This will be explained in more detail in the next subsection.

Figure 9 shows that the crystallinity of the multilayer samples was higher than that for the PETg sheet produced from virgin pellets, even though the multilayer samples contained only a fraction of PETg. This means that crystallization was promoted in the multilayers. Furthermore, the degradation had an influence on the crystallinity. Sheet ‘2080’ contained less PETg than ‘ML’, and ‘5050’ contained less PETg than ‘2080’, yet their crystallinity was comparable. This means that the recycling step caused the PETg regions to become more crystalline. This might be due to the chain scission induced by regrinding and reprocessing, leading to shorter chains, which facilitates crystallization [41,42,43,44].

It can also be noted that the crystallinity was reduced due to the thermoforming process. This could have been caused by the fact that spherulites are torn apart during stretching. This effect was also observed by Makradi et al. [46] for the thermoforming of isotactic polypropylene (iPP). It was also found that PETg is not very prone to strain-induced crystallization [47].

### 4.6. Optical Properties of the Sheet and the Thermoformed Tray: Haziness and Yellowness Index

**Figure 10:**
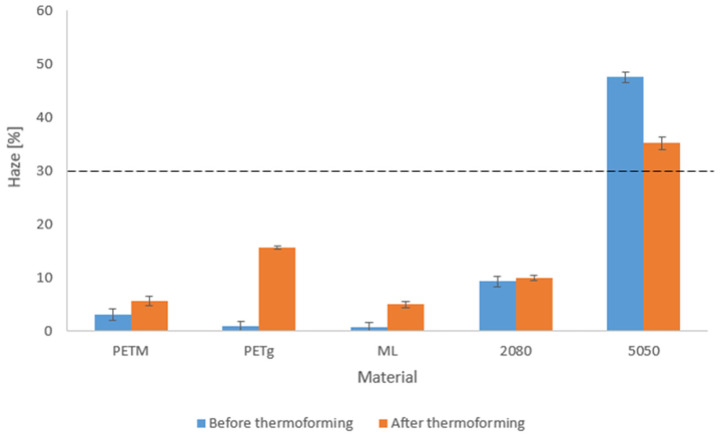
Haze of the sheets, extruded with the Labtech co-extrusion unit, and the thermoformed trays.

In Figure 10, the haze of the sheets and trays is shown. The thicknesses of both can be found in Table 4. The thickness should be taken into account when comparing sheets and trays. The trays were less thick, which means that if thermoforming had no effect on the haze, the haze should decrease, since there is less chance of light hitting defects which could cause scattering as light passes through the sheet (Lin et al.) [48]. Another aspect which is important to take note of is that the surface of the tray was slightly ribbed. This could also cause some scattering (Lin et al., Maruhashi et al.) [48,49]. Once the haze percentage reaches 30%, the material is no longer defined as transparent, but as translucent [48].

From Figure 10 can be deduced that the haze of PETM was not less than 1%, which was suggested on the datasheet. Since PETM is totally amorphous, the haze of this sample could not be caused by the crystallinity of the material. Since the sheet and tray only contained one material, improper blending had no effect and this haziness must have been caused by voids. This means that the drying step before processing was insufficient and caused evaporation inside the barrel, leading to voids inside the sample that scattered light. This was confirmed by measurements which showed a residual moisture content of 0.13%. The haziness was somewhat larger after thermoforming. This might be caused by the fact that the voids were stretched, which makes them larger in the stretch direction and leads to more light scattering. This effect was also seen by Maruhashi et al. and Prattipati et al. [49,50]. The ribbed structure of the tray can also have an impact.

For the PETg sheet, a haze of less than 1% was measured. It is probable that the PETg was dried better before processing than was the case for PETM. This was confirmed by measurements which showed that the residual moisture content in the PETg pellets was 0.07%. Since PETg is a crystalline material, higher temperatures could be used for drying, which was certainly an advantage. Although the thickness of the tray was less than that for the sheet, the haziness of the tray was significantly higher. Thus, thermoforming leads to additional haze in PETg trays. DSC results showed that this additional haze was not caused by an increase in crystallinity. The ribs of the tray could have an impact; however, this could not be the main cause, since the haze was significantly more increased than it was for the PETM trays. Lin et al. [48] described the creation of micro-voids during thermoforming, which lead to additional haze due to the fact that they are able to scatter light.

The DSC data showed that all multilayer sheets had a comparable crystallinity (Figure 9). However, the haziness of the sheets was much larger with higher amounts of regrind. The virgin multilayer showed promising results, with its haziness of less than 1%. The worse results for the multilayers containing regrind could be due to insufficient drying of the regrind, since the same temperatures were used as for PETM. Measurements showed a residual moisture content of 0.11%. A second possibility is that the PETM and PETg fractions were not totally mixed, leading to small regions of one material encapsulated inside the other, scattering light. Both explanations might have a relevant impact, since the haze was significantly higher at higher amounts of regrind. For the ‘ML’ and ‘2080’ multilayers, the haziness rose after thermoforming, although the thickness decreased. This could not be caused by cold crystallization of the PETg fraction, since no increase in crystallinity was detected in the DSC measurements. Since almost no haze was detected in the ‘ML’ sheet, the additional haze in the tray could only be caused by micro-voiding or the effect of the tray ribs, whereas for the ‘2080’ tray the stretching of already existing voids could also have had an effect. For the ‘5050’ tray, the haze exceeded 30%, making the material translucent, and it is evident that the haze decreased after thermoforming. It is possible that no additional micro-voids were formed, since there was already a large number of voids present in the sample, and only the already existing ones were stretched.

**Figure 11:**
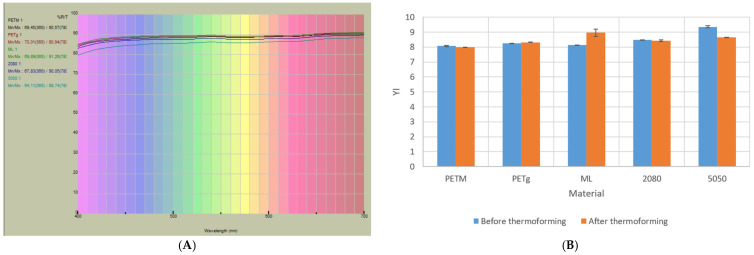
(**A**) VIS spectrum of sheets and trays. (**B**) Yellowness index values of the sheets (blue) and thermoformed trays (orange) (Table 2).

For all samples, a VIS spectrum was measured. These looked similar for all samples and showed that the sheets absorbed light in the violet region (Figure 11A). This implies that the sheets show a certain yellowness. The YIs were calculated and are presented in Figure 11B. It can be seen that the YI was around eight for all the sheets and trays. This is still not visible with the naked eye. For the multilayer sheets, the YI becomes slightly higher with rising regrind fraction (4% for 20% and 13% for 50% regrind in the middle layer). This could be expected, since the regrind fraction has gone through two processing steps, leading to more degradation. It was also evident that the YI increased for the ‘ML’ sheet. However, this was not visible for the other sheets, and the reverse could be noticed for the ‘5050’ sheet.

### 4.7. Resistance against Deformation during Hot Fill of the Thermoformed Trays

**Figure 12:**
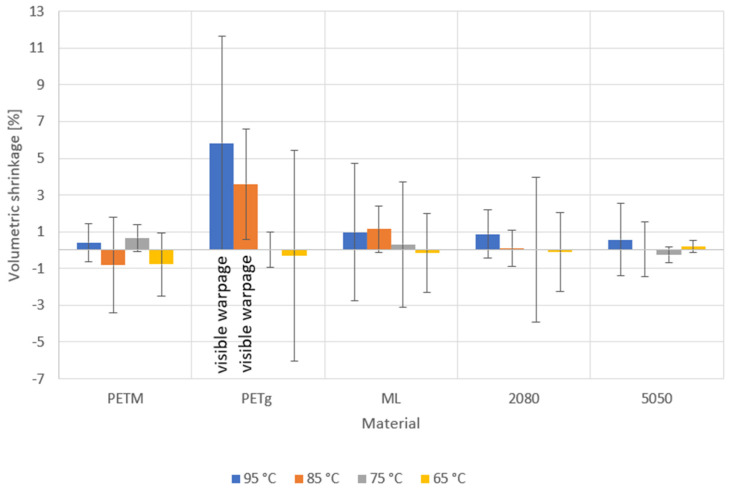
Volumetric shrinkage of the trays from different materials (Table 2) after hot filling for the different initial hot-fill temperatures. The 95% confidence interval of the volumetric shrinkage is given by the error bars.

The hot-fill test was carried out at four different temperatures. During testing, the temperature of the water inside the trays was monitored. These temperatures can be found in Table 5. The measured shrinkage is shown in Figure 12.

Some results show a negative shrinkage after hot filling (Figure 12). This implies that the fault on the measurements cannot be neglected. To see if the change in volume is statistically relevant, two statistical methods were used.

**Figure 13:**
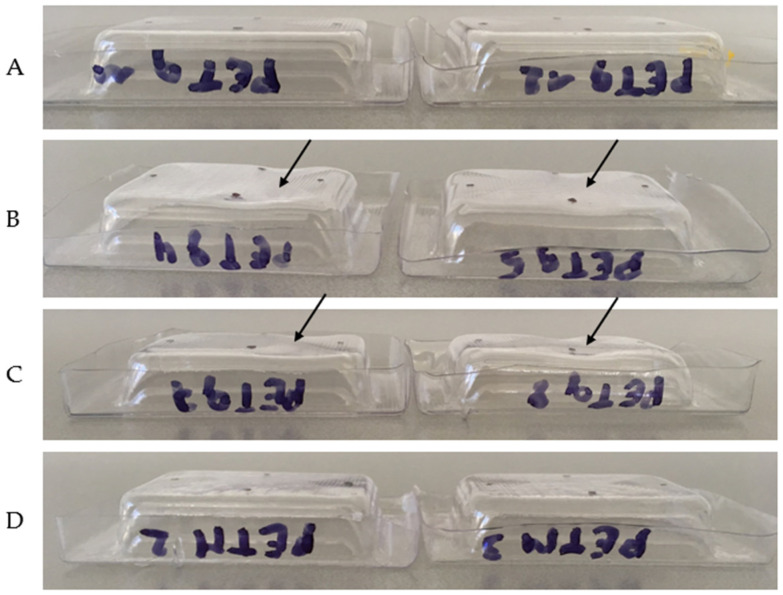
PETg trays exposed to hot-fill temperatures of 75 °C (**A**), 85 °C (**B**) and 95 °C (**C**), and PETM trays exposed to hot-fill temperatures of 95 °C (**D**).

First of all, the volume of a certain tray was measured 12 times, spread over two days. The standard deviation was found to be 0.2307 mL. This means that the 95% confidence interval of the true volume of a measured tray lay between the measured value ±0.4614 mL. Thus, if the difference between the measurements before and after hot filling was larger than 0.9227, this means that the tray had changed in volume with a certainty of 95%. This was only the case for the PETg trays which were exposed to temperatures of 95 °C and 85 °C. For these trays, the warpage was visible to the naked eye (Figure 13B,C).

A second statistical testing procedure was conducted by calculating the percentual change in volume of two samples which were exposed to the same circumstances. Afterwards, the SPSS software package was used to execute a one-sample *t*-test and to determine whether these values indicated that the population differed from zero with a certainty of 95, since this would indicate that no change in volume had occurred. Again, only for the PETg trays exposed to 95 °C and 85 °C was the difference between the measured values and zero statistically relevant. Moreover, the difference between the shrinkage at 85 °C and 95 °C was statistically significant as well, meaning that higher temperatures lead to more shrinkage.

These tests showed that the outer PETM layer with the total thickness of 1 mm, being 10% of the total sheet thickness, is sufficient to preserve the PETg middle layer (or partially recycled layer) from deforming, which makes the multilayer material thermostable enough for hot-fill applications.

## 5. Conclusions

In this study, we explored the potential of PETM, a new modified PET material, for the production of hot-fill-based food packaging. For comparison, we also selected PETg. We first focused on extrusion-based insights and then on thermoforming and actual hot-fill results.

We addressed the processability and extrusion-induced degradation of the PETM and PETg polymers through FTIR and inherent viscosity measurements. It was shown that higher residence times did not result in more significant degradation. The shorter chains also influenced the OIT, which decreased due to the higher mobility and reactivity of the shorter chains. The thermal degradation seemed to occur at higher temperatures for longer residence times, which might be caused by the formation of stable oxidation products. The thermal stability of PETM was found to be higher than for PETg.

We also produced trays from PETg, PETM and multilayer sheets. Thermoforming did not cause notable degradation of the sheets. However, a decrease in crystallinity was detected, which was probably due to tearing of the spherulites. The PETg and multilayer trays produced from virgin material showed good clarity (<1% haze). For PETM, the measured haze was higher than expected (±5%), probably caused by voids which were present due to the evaporation of residual water during processing. For the sheets containing regrind material, higher hazes of about 9% and 47.5% were measured, probably due to bad mixing of the blend and the presence of moist during processing. Determination of the yellowness index showed that all sheets had a YI of approximately eight. No clear effect of thermoforming could be found.

The influence of hot fill was tested by measuring the dimensional stability of the hot-filled tray. It was shown that the multilayer material does not deform during the hot fill process, whereas the PETg tray could not withstand filling temperatures of 95 °C or 85 °C.

We have thus shown that PETM-based multilayers have great potential for hot-fill applications within the food industry, making them very promising from an application point of view. Moreover, the multilayers show great recycling potential. There is no doubt that these factors will contribute to raising the commercial potential of such materials. Clear trays with a sufficient thermal stability were obtained, although some haziness occurred when regrind was added to the middle layer. Thus, the developed multilayer material exhibits (1) high thermal stability, making it applicable for hot-fill food packaging applications; (2) high transparency; and (3) excellent recyclability, as the addition of the regrind does not affect its thermal or optical properties.

## Figures and Tables

**Table 1:** Barrel temperature, screw speed and (total) residence time of the residence-time samples of PETM and PETg.

Sample	Barrel Temperature (°C)	Screw Speed (rpm)	Added Residence Time (min)	Total Residence Time (min)
PETM 1	270	60	2	6
PETM 2			5	9
PETM 3			8	12
PETM 4			11	15
PETg 1	290		2	6
PETg 2			5	9
PETg 3			8	12
PETg 4			11	15

**Table 2:** Temperatures inside the LabTech co-extrusion unit during sheet extrusion, in which E1 (screw diameter of 30 mm) extrudes the middle layer, E2 extrudes the first outer layer (screw diameter of 30 mm) and E3 extrudes the other outer layer (screw diameter of 20 mm).

Sample	E1	E2	E3	Die
PETM	240–250–260–280–280 °C 180 rpm	240–250–260–270–270 °C 10 rpm	260–265–270–270 °C 40 rpm	280 °C
PETg	260–270–280–290–290 °C 48 rpm	260–270–280–290–290 °C 48 rpm	280–285–290–290 °C 67 rpm	280–290–280 °C
ML	260–270–280–290–290 °C 85 rpm	260–260–265–270–270 °C 11 rpm	260–265–270–270 °C 43 rpm	280–290–280 °C
2080	260–270–280–290–290 °C 80 rpm	260–260–265–270–270 °C 10 rpm	260–265–270–270 °C 40 rpm	280–290–280 °C
5050	260–270–280–290–290 °C 80 rpm	260–260–265–270–270 °C 10 rpm	260–265–270–270 °C 40 rpm	280–290–280 °C

**Table 3:** Useful temperature range for thermoforming the PETM, PETg and multilayer sheets.

Sample	Temperature Range
PETM	155 °C–165 °C
PETg	135 °C–142 °C
ML	145 °C–150 °C
2080	145 °C–150 °C
5050	145 °C–153 °C

**Table 4:** Thicknesses of the extruded sheets, and the thermoformed trays.

Sample (Table 2)	Thickness of Sheet (mm)	Thickness of Tray (mm)
PETM	1.010	0.584
PETg	0.948	0.680
ML	1.016	0.800
2080	1.038	0.793
5050	1.037	0.788

**Table 5:** Temperatures of the water inside the tray after 1 and 2.5 min of hot fill for the different initial hot-fill temperatures.

Filling Temperature (°C)	Temperature after 1 min (°C)	Temperature after 2.5 min (°C)
95	85	78
85	75	70
75	65	63
65	60	55
